# An Investigation on the Quantitative Structure-Activity Relationships of the Anti-Inflammatory Activity of Diterpenoid Alkaloids

**DOI:** 10.3390/molecules22030363

**Published:** 2017-02-27

**Authors:** Xiao Li, Ning Li, Zhenyu Sui, Kaishun Bi, Zuojing Li

**Affiliations:** 1 School of Pharmacy, Shenyang Pharmaceutical University, Shenyang 110016, China; xiao6615@163.com (X.L.); iamdanielsui@126.com (Z.S.); kaishunbi.syphu@gmail.com (K.B.); 2 School of Chinese Traditional Materia Medica, Shenyang Pharmaceutical University, Shenyang 110016, China; liningsypharm@163.com; 3 School of Medical Devices, Shenyang Pharmaceutical University, Shenyang 110016, China

**Keywords:** diterpenoid alkaloids, anti-inflammatory, quantitative structure-activity relationship (QSAR)

## Abstract

Diterpenoid alkaloids are extracted from plants. These compounds have broad biological activities, including effects on the cardiovascular system, anti-inflammatory and analgesic actions, and anti-tumor activity. The anti-inflammatory activity was determined by carrageenan-induced rat paw edema and experimental trauma in rats. The number of studies focused on the determination, quantitation and pharmacological properties of these alkaloids has increased dramatically during the past few years. In this work we built a dataset composed of 15 diterpenoid alkaloid compounds with diverse structures, of which 11 compounds were included in the training set and the remaining compounds were included in the test set. The quantitative chemistry parameters of the 15 diterpenoid alkaloids compound were calculated using the HyperChem software, and the quantitative structure–activity relationship (QSAR) of these diterpenoid alkaloid compounds were assessed in an anti-inflammation model based on half maximal effective concentration (*EC*_50_) measurements obtained from rat paw edema data. The QSAR prediction model is as follows: $\log\left(EC_{50}\right)=-0.0260\times \mathrm{SAA}+0.0086\times \mathrm{SAG}+0.0011\times \mathrm{VOL}-0.0641\times \mathrm{HE}-0.2628\times \mathrm{LogP}-0.5594\times \mathrm{REF}-0.2211\times \mathrm{POL}-0.1964\times \mathrm{MASS}+0.088\times \mathrm{BE}+0.1398\times \mathrm{HF}$ (*R*^2^ = 0.981, *Q*^2^ = 0.92). The validated consensus *EC*_50_ for the QSAR model, developed from the rat paw edema anti-inflammation model used in this study, indicate that this model was capable of effective prediction and can be used as a reliable computational predictor of diterpenoid alkaloid activity.

## 1. Introduction

The diterpenoid alkaloids are found in many traditional herbal medicines (TCM), such as Consolida, Aconitum, and Delphinium. Pharmacological studies have indicated that many diterpenoid alkaloids are important chemical constituents of this herbal remedies with analgesic, anti-cancer, anti-inflammatory, and anti-arrhythmic activities [1]. The anti-inflammatory activity was the first confirmed important pharmacological activity of the diterpenoid alkaloids, and is stronger than that of many active anti-inflammatory drugs [2].

Most of the diterpenoid alkaloids are isolated from *Aconitum, Consolida* and *Delphinium* species, which have been widely used in medicine with excellent clinical efficacy for thousands of years. There are three categories of diterpenoid alkaloids containing 18, 19 and 20 carbon atoms, respectively, which are classified as the C18-, C19-, and C20-diterpenoid alkaloids [3]. Different types of diterpenoid alkaloids show diverse biological activities, for example, C18-, C19-diterpenoid alkaloids exhibit anti-inflammatory and analgesic activities [4], while C20-diterpenoid alkaloids possess potent inhibitory activity on tumor cells [5].

The two main types of analgesic and anti-inflammatory drugs are non-steroidal anti-inflammatory drugs (NSAIDS) and opioids. However, NSAIDS might cause gastrointestinal tract damage, while opioids are harmful to the nervous system and have a strong potential for causing addiction [6,7]. This makes it necessary to develop new types of analgesic and anti-inflammatory drug with lower toxicity and higher anti-inflammatory activity. Although diterpenoid alkaloids are toxic, they have significant pharmacological activities. Diterpenoid alkaloids interact with receptors in the neurotransmitter systems and have electrophysiological properties, therefore are the good candidates to develop drugs with anti-inflammatory activity [2]. The relationship between the structure and activity is determined by the structure-activity relationship, in order to modify the structure of the compounds and enhance the anti-inflammatory activity [2], hence we studied the relationship between the activity and structure of the diterpenoid alkaloids.

Recent studies have proved that some diterpenoid alkaloids have strong anti-inflammatory activities [8]. They significantly inhibited rat paw edema caused by a variety of inflammatory agents, such as egg white, carrageenan, histamine and 5-HT; the xylene-induced mouse ear edema; the histamine, 5-HT-induced capillary permeability increase; and reduced the inflammatory exudates [9,10,11]. In this paper, the anti-inflammatory activity of the diterpenoid alkaloids in the rat paw edema inflammation model was investigated.

Quantitative structure activity relationship (QSAR) has been demonstrated as a useful tool for investigating the bioactivities of various classes of compounds. The purpose is to find the relation between the composition or structure of a compound and its chemical activity [12,13,14]. It can be further used to guide chemical synthesis when new chemical entities are developed. Projections to latent structures represent a regression technique for modeling the relationship between projections of dependent factors and independent responses [15]. Partial least squares (PLS) regression, a multivariate regression modeling method, can be applied to conditions in which the number of independent variables is greater than the number of observations [16]. The observation can be projected from a high-dimensional space to a low-dimensional space to reduce the number of model input variables [17]. The PLS approach is a statistical modeling technique with data analysis features linking a block (or a column) of response variables to a block of explanatory variables [18]. It leads to stable, correct and highly predictive models even for correlated descriptors [19]. The PLS method is widely used in measuring chemical information and biological information, as well as in other fields. It has recently been applied to study QSTR of toxic compounds and yielded good results [20]. In this paper, a QSAR study was conducted on 15 diterpenoid alkaloids, analyzed by Hyperchem, to obtain the molecular structure parameters and establish a predictive model of *EC*_50_ measurement in rat paw edema using the PLS method.

## 2. Results and Discussion

### 2.1. Modeling

**Figure 1:**
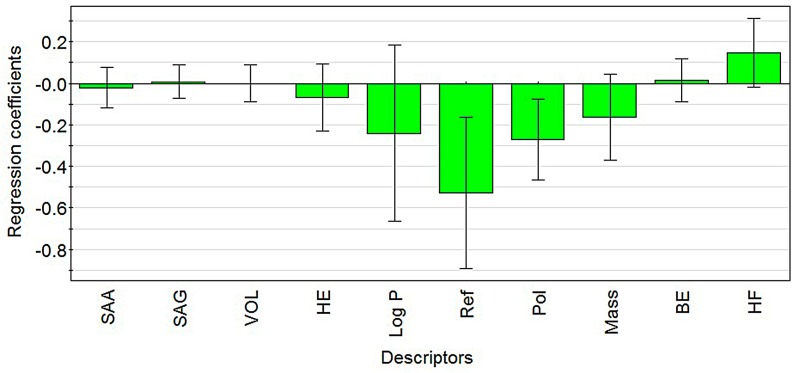
The regression coefficient diagram of descriptors in the QSAR model.

In this study, the PLS method was applied to build the QSAR model between the quantitative 3D structural parameters of various diterpenoid alkaloid compounds and the rat paw edema anti-inflammation model activities, due to the fact that diterpenoid alkaloids are natural ingredients isolated from TCM, and we had only a small number of compounds. The activity of several diterpenoid alkaloids was predicted using the model, which showed good correlation and predictive ability. The relationship between Log(*EC*_50_) and 10 variables modeled by PLS was as follows:

(1) $$\log\left(EC_{50}\right)=-0.0260\times \mathrm{SAA}+0.0086\times \mathrm{SAG}+0.0011\times \mathrm{VOL}-0.0641\times \mathrm{HE}-0.2628\times \mathrm{LogP}-0.5594\times \mathrm{REF}-0.2211\times \mathrm{POL}-0.1964\times \mathrm{MASS}+0.088\times \mathrm{BE}+0.1398\times \mathrm{HF}$$

where SAA is surface area approx., SAG is surface area grid, VOL is volume, HE is hydration energy, REF is refractivity, POL is polarizability, BE is binding energy, and HF is heat of formation. The regression coefficient diagram is presented in Figure 1 to intuitively explain the activity values of the diterpenoid compounds.

**Figure 2:**
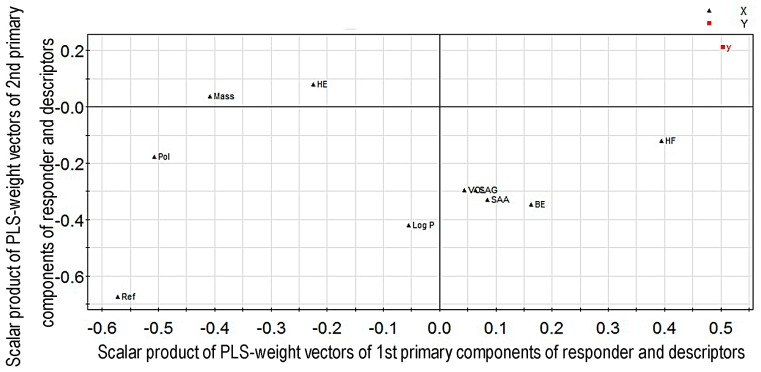
The loading plot of PLS-weight vectors for the first two primary components in the QSAR model (W*C).

From the regression coefficient diagram (Figure 1), we conclude that the value of *EC*_50_ increased when the variables including surface area grid, volume, hydration energy, heat of formation and binding energy increased, and the value of *EC*_50_ decreased when the variables including surface area approx., LogP, mass, polarizability and refractivity increased. Greater value of *EC*_50_ indicates lower activity of the compound. Thus, surface area approx., LogP, mass, polarizability and refractivity were positively related to the activity of diterpenoid alkaloid compounds, while surface area grid, volume, hydration energy, heat of formation and binding energy were negatively correlated with diterpenoid alkaloid compounds. The loading plot of PLS-weight (the PLS analysis results in model coefficients of the latent variables) vectors for the first two primary components is shown in Figure 2, which is the scalar product of responders and descriptors. The weights for the descriptors, denoted by “W”, indicate the importance of these variables and how much they “in a relative sense” participate in the modeling of the responder. The weights for the responders, denoted by “C”, indicate which responder is modeled in the respective PLS model dimensions (Table S1 in the Supplementary Material section). Therefore, the farther a point (i.e., variable) locates away from the origin, the more it contributes to PLS model prediction.

**Figure 3:**
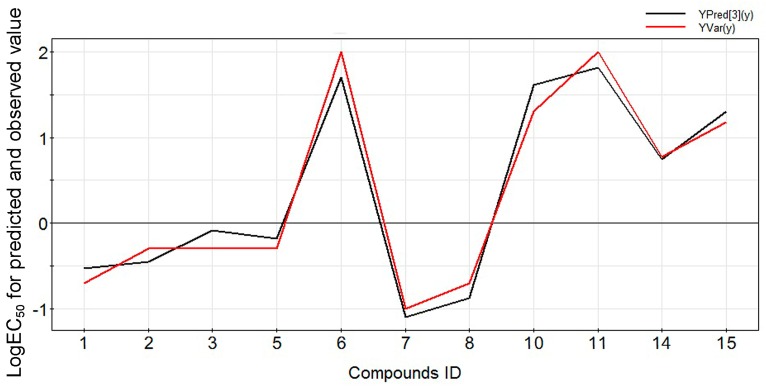
The curve fitting graph for the training dataset. YPred[3](y) and YVar(y) are respectively the predicted and observed values for the training set.

Based on the regression coefficient diagram (Figure 1), the contributions in the decreasing order, for PLS model prediction among descriptors in QASR, is ReF, LogP, POL, MASS, HF, BE, HE, SAA, SAG, and VOL, consistent with the conclusion drawn based on the regression coefficient diagram of descriptors in the QSAR model (Figure 1). Furthermore, the curve fitting graph for the predicted and observed values of the activities of the diterpenoid alkaloids is shown as Figure 3, indicating a good fitting result.

**Figure 4:**
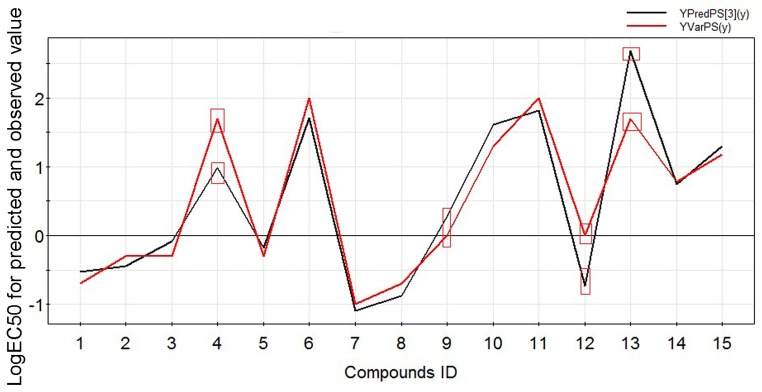
The comparison between the predicted and observed values (compound ID **4**, **9**, **12**, **13** as the test set, and others as the training set).

The predictive ability of the model was tested using four other diterpenoid alkaloids (compound IDs **4**, **9**, **12**, **13**) as a forecasting dataset (Table 1 and Figure 4). The prediction correlation coefficient was *R*^2^*_pred_* = 0.8320 > 0.5 (Table 1), which reflected the statistical significance of the model. In addition, *Q*^2^ was greater than 0.5, indicating good predictive ability of the PLS model.

**Figure 5:**
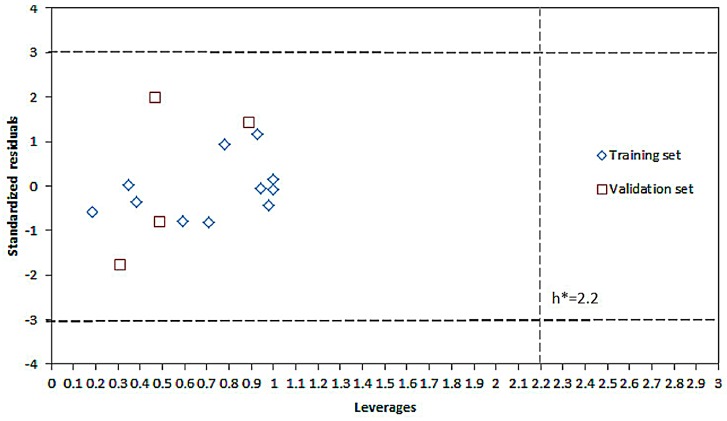
Williams Plot: standardized residuals versus leverages, horizontal dash lines indicate ±3 SD unites, vertical dash line indicates the threshold value h* = 2.2).

The applicability domain (AD) of the QSAR model was characterized by the Williams plot as Figure 5 [14,21,22].

The leverage values for all compounds were lower than critical value, and the prediction errors for all compounds were between ±3 SD unites. Thus, all compounds studied belong to the AD of the QSAR model.

### 2.2. Experimental Verification for Model Accuracy

In order to verify the prediction accuracy of the PLS model introduced in Section 2.1, model accuracy verification was carried out. The anti-inflammatory activities for three diterpenoid alkaloids including delsoline, fuziline and songorine have been predicted by the PLS QSAR model (Equation (1)). The model-predicted Log(*EC*_50_) for songorine was 0.301, and were 4.0517 and 3.8805 for delsoline and fuziline, which do not indicate activities for fuziline and delsoline since Greater value of *EC*_50_ indicates lower activity of the compound. (Table S2 in the Supplementary Materials).

**Figure 6:**
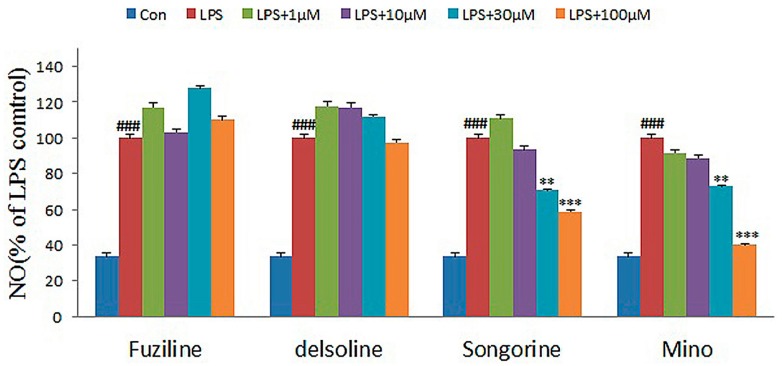
Anti-inflammatory activity assay of the compounds (Fuziline, delsoline, Songorine and Mino) in N9 microglial cells. Each bar represents the mean ± SE of three independent experiments. One-way ANOVA adjusted by Dunnett’s test was used for statistical analysis (SPSS 15.0 software, SPSS). ### *p* < 0.001 compared with control group, ** *p* < 0.01 and *** *p* < 0.001 compared with LPS group.

To determine whether they have potential anti-inflammatory activities, the effects of three unknown compounds on the release of nitric oxide (NO) were determined using microglial cell line (N9) activated by lipopolysaccharide (LPS). Minocycline, a known anti-inflammatory compound, was used as the positive control. The results are shown in Table 2 and Figure 6.

As shown in Figure 5, songorine inhibited the production of NO in LPS-activated N9 cells in a dose-dependent manner without obvious cytotoxicities, suggesting that it might be a potential inflammatory inhibitor, consistent with the model prediction (Log(*EC*_50_) = 0.301). However, fuziline and delsoline didn’t exhibit inhibitory effects at the tested concentrations, consistent with the model predicted value.

### 2.3. Molecular Docking

**Figure 7:**
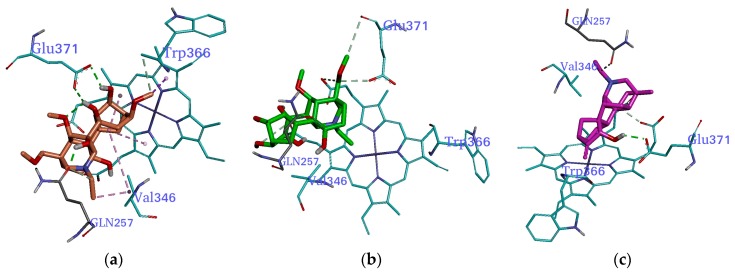
Interaction modes of delsoline, fuziline and songorine within iNOS binding pocket. (**a**) H-bonds between delsoline and iNOS pocket; (**b**) H-bonds between fuziline and iNOS pocket; (**c**) H-bonds between songorine and iNOS pocket.

In order to clarify the anti-inflammatory activity of delsoline, fuziline and songorine, molecular docking study was conducted to measure the relative binding energies and localize binding sites in iNOS, shown in Figure 7. Binding free energy evaluations were used to find the best conformation binding mode of ligand position in AutoDock 4.0. The final binding free energy is based on intermolecular energy, internal energy of ligand, and torsional free energy [23]. The potential energy was minimized for the best ranked docking pose. For the top ranked poses, the calculated binding energy of iNOS complexed with delsoline, fuziline and songorine were −6.48, −7.08 and −9.82 kcal/mol, respectively.

The bright green dashed lines represent the typical hydrogen bond. The binding mode between compounds delsoline, fuziline, songorine and iNOS was compared at Val-346, Trp-366, Gln-257 and Glu-371. The more typical hydrogen bonds, the better binding affinity between the compound and iNOS. The binding affinity for delsoline-receptor interaction was the best, followed by songorine-receptor interaction and fuziline-receptor interaction (Figure 7). From a thermodynamic point of view, a negative free energy (ΔG < 0) indicates a favorable stable system [24]. High affinity between iNOS and songorine was observed, consistent with high affinity between iNOS and its classic substrate fuziline, suggesting that songorine most likely has anti-inflammatory activity. The estimated values in the molecular docking study were almost equal to the model prediction results.

In recent years, natural products have attracted a lot of attention. Many compounds have been isolated from natural products and confirmed to possess excellent pharmacological efficacies. Various pharmacological activities of the diterpenoid alkaloids, including analgesic, anti-inflammatory, antimicrobial, antitumor, cardiotonic and anti-arrhythmic activities have been discovered. Inflammation is a defense reaction of the body. The traditional anti-inflammatory drugs sometimes cannot be applied to disease treatment because of their side effects. We have to find new anti-inflammatory compounds and there is a need to discover new analysis methods to study the relationships between activity and structure. QSAR is a useful tool to analyze relationships between anti-inflammatory activities and structure using PLS. The reliable model should help us with the prediction of biological activities for newly designed compounds.

## 3. Materials and Methods

### 3.1. Data Description

**Figure 8:**
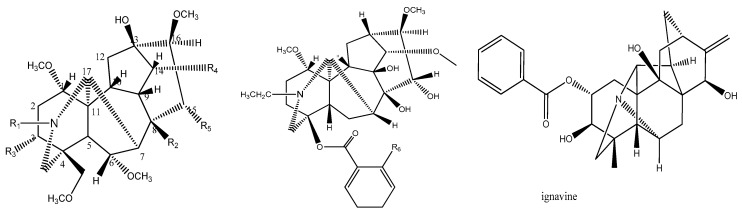
The chemical structures of 15 diterpenoid alkaloids.

The activity values of 15 diterpenoid alkaloids (structures shown in Figure 8 and Table 1) from the rat paw edema inflammation model were obtained from published literatures [25,26] (Table 3). 3D structure parameters of 15 compounds were determined using Hyperchem software, including surface area approx. (SAA), surface area grid (SAG), volume (VOL), polarizability (POL), hydration energy (HE), mass, LogP, refractivity (REF), heat of formation (HF) and binding energy (BE). A conformational analysis of the molecules was performed using ‘‘optimal search method’’ prior to the calculation of 3D descriptors. The chemical structures of 15 kinds of diterpenoid alkaloids compounds were entered into hyperchem program software and were optimized using geometry optimizing by their gradient which was less than 0.10, and were imaged by three dimensional formations. Then all kinds of quantitative three dimensional parameters of diterpenoid alkaloids compounds were calculated using semiempirical formula method [27]. What is also worth considering is that HF was obtained using PM3 quantum chemical calculations and other parameters were obtained using AM1 quantum chemical calculations. A model was established with these parameters as independent variables using 11 compounds as a training set for modeling. The PLS method was used to establish the QSAR model between the activity and structure of these compounds [28].

**Figure 9:**
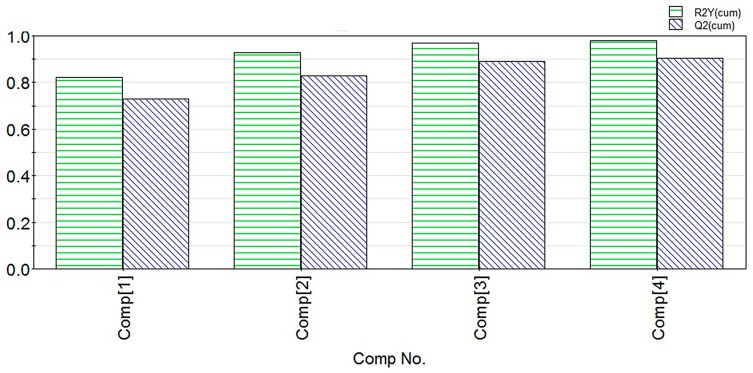
*R*^2^ and *Q*^2^ parameters compared with **1**–**4** components extracted.

The QSAR model was built based on the PLS method using the SIMCA-P software [29]. Briefly the number of PLS component extraction was selected and determined based on the change of the parameters. The number of components was selected as **1**, **2**, **3** and **4**, respectively. The parameter *R*^2^ for the explanatory ability of the model and the cross validation parameter *Q*^2^ calculated with different PLS extraction components are shown in Figure 9.

A logarithm transformation was used to process the *EC*_50_ values since the values showed wide variations, and the value of hydration energy was reduced by 100-fold for the same reason.

**Figure 10:**
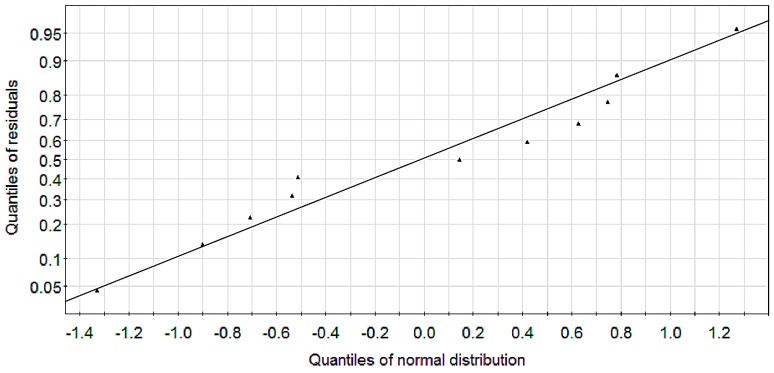
PLS-QSAR residual scatter.

**Figure 11:**
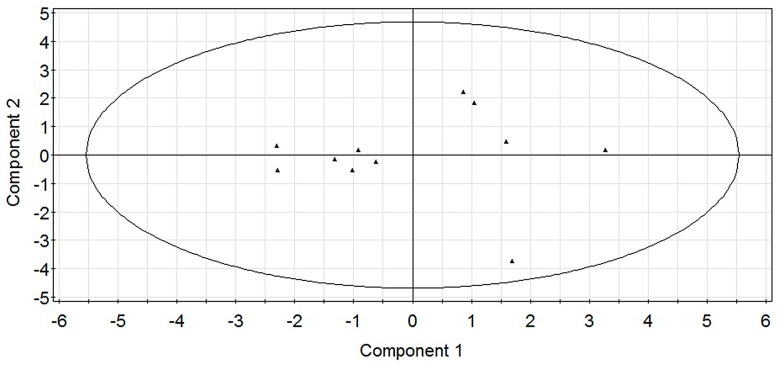
PLS-QSAR score plot.

When four PLS components were extracted, the values of *R*^2^ and *Q*^2^ increased gradually (Figure 9). Three components were exacted because *R*^2^ that represents the explanatory ability of the model was close to 1 and the cross validation parameter *Q*^2^ was greater than 0.5, with the coefficient of determination was *R*^2^ = 0.981 and the leave-one-out cross validation was *Q*^2^ = 0.925. *R*^2^ and *Q*^2^ had no significant changes when four components were extracted. When the statistical parameters *R*^2^ > 0.600 and *Q*^2^ > 0.500, the model is effective [30,31,32,33]. Therefore, we extracted three components to build the model, and drew the residual scatter and score plot (Figure 10 and Figure 11).

As shown in Figure 10, residuals can be treated as normally distributed since all points lied closely to a straight line. As shown in Figure 11, sample points were distributed within the ellipse, indicating no singular points and good model.

The predictive ability of the model was examined through prediction correlation coefficient *R*^2^*_pred_* [34], which is calculated according to the following equation:

(2) $${R^{2}}_{pred}=1-\frac{\sum {\left(Y_{pred(test)}-Y_{\left(test\right)}\right)}^{2}}{\sum {\left(Y_{\left(test\right)}-{\bar{Y}}_{training}\right)}^{2}}$$

where *Y_pred_*_(*test*)_ and *Y*_(*test*)_ is the predicted and observed *EC*_50_ values of the test set, respectively; and training is the mean *EC*_50_ value of the training set. The value of *R*^2^*_pred_* should be greater than 0.5.

The application domain (AD) of the model was validated by the Williams plot of standardized residuals versus leverage values (*h^i^*) compared with a critical value (h*) [21], which is calculated according to the following equation:

(3) $$h^{i}={x_{i}}^{T}{\left(X^{T}X\right)}^{-1}x_{i}$$

(4) $$h^{*}=3\left(k+1\right)/n$$

where *x_i_* is the descriptor vector of the compounds, *X* is matrix of descriptors, *k* is the number of descriptors and *n* is the number of objects used to calculate the model. When the absolute standardized residual for a compound exceed three standard deviation units, the compound is outside the AD of the model; if the leverage value of a compound is greater than the critical value, molecular structure could influence model, so the model is not reliable [14,21,22].

### 3.2. Experiments Methods

#### 3.2.1. Cell Culture

The murine microglial cell line N9 cells [35,36,37,38,39,40] were cultured in DMEM supplemented with 10% FBS, 2 mM glutamine, 100 U/mL penicillin, and 100 μg/mL streptomycin at 37 °C in humidified 5% CO_2_.

#### 3.3.2. Sample Preparation

Fuziline, delsoline and songorine were purchased from Chengdu Must Bio-technology Company (Chengdu, China) with purity assayed by HPLC at 98.00%, 98.00% and 99.91% respectively. These compounds in powder form were dissolved in DMSO at 100 mM and stored at −20 °C. Stock solution was diluted to 100 μM, 30 μM, 10 μM, and 1 μM using IMDM for the experiment.

#### 3.2.3. Measurement of Cell Viability

Before nitrite assay, cell viability was measured with MTT assay [37,38,39,40,41,42]. N9 cells were seeded into 96-well plates, incubated with the tested compounds at 100 μM, 30 μM , 10 μM and 1 μM in the presence of LPS (100 ng/mL) for 24 h, then treated with 0.25 mg/mL MTT at 37 °C for 4 h. After the supernatant was removed, the formazan crystals produced by MTT reduction were dissolved by adding DMSO (100 μL). Then the proportion of viable cells was determined by colorimetric assay in a plate reader (Bio-Tek, Winooski, VT, USA) at 490 nm.

#### 3.2.4. Nitrite Assay

The level of nitrite (NO^2−^) in the supernatant of cultured cells was determined by Griess assay [35,36,37,38,39]. N9 cells in 96-well plates were treated with the tested compounds at 100 μM,30 μM, 10 μM and 1 μM in the presence of LPS (100 ng/mL) for 24 h. 50 μL supernatant was then added to 50 μL Griess reagent and incubated for 15 min at room temperature. Finally, the absorbance of the tested samples was determined with a plate reader (Bio-Tek) at 540 nm.

#### 3.2.5. Anti-Inflammatory Assay in N9 Microglial Cells

The activation of microglia plays an important role in the development of chronic inflammation-mediated neurodegenerative diseases. Inhibiting the activity of microglia may become a new target for drug discovery. The microglia cells are activated by LPS and release NO, inflammatory cytokines and ROS. In this study, an in vitro screening model was established using the LPS-induced abnormal activation of N9 microglia cells and NO release as an indicator

- Griess assay inhibitory effects of compounds using N9 microglial cells

N9 microglia cells in the logarithmic phase were cultured for 24 h and then incubated with the compounds at 100 μM, 30 μM, 10 μM, and 1 μM respectively, with the combined action of LPS at 1000 ng/mL. For blank control group, cells didn’t receive any compound or LPS treatment. Cells were cultured for another 24 h. Cell culture media was collected and the Griess colorimetry was used to detect NO content in the media.

- Measurement of cell viability

Cell viability was examined by the MTT reduction assay. N9 cells were seeded into 96-well plates were incubated with 20 μL/well MTT solution for 4 h at 37 °C. Culture media was collected, 150 μL DMSO was added, and the optical density OD value was measured. The average of OD values of each sample was used to calculate the cell survival rate (CV %) according to the following formula:

Cell survival rate % = average OD value of sample/average OD value of blank control group × 100%(5)

#### 3.2.6. Molecular Docking

Molecular docking is a powerful tool in structural molecular biology and computer-assisted drug design. Computer programs dedicated to docking small molecules into protein binding pockets are currently a focus of attention for many research groups [23,40,41]. Inducible arginine oxidation and subsequent NO production by correspondent synthase (iNOS) are important cellular answers to pro-inflammatory signals, and selective iNOS inhibitors may be useful as anti-inflammatory drugs [42].

In this study, AutoDock4.0 was used for molecular docking study. The crystal structure of iNOS in complex with a nanomolar imidazopyridine inhibitor (PDB code 3NW2) [43] was used. The co-crystal ligand was extracted to define active site, and polar hydrogen atoms were added to protein geometrically. The docking area was assigned around the active site with AutoDock Tools (ADT) [43]. A grid of 30 Å × 30 Å × 30 Å with 0.175 Å spacing was calculated around the docking area for ligand atom types using AutoGrid. Nine three docking calculations were performed for both delsoline, fuziline and resveratrol, as each docking calculation consisted of 25 million energy evaluations using Lamarckian genetic algorithm local search method, with a population size of 200, and 3000 rounds of Solis and Wets local search. The docking results from each of the nine calculations were clustered on the basis of root-mean-square deviation (rmsd) and were ranked on the basis of free binding energy. The top-ranked compounds were visually inspected with Accelrys Discovery Studio Visualizer [35].

## 4. Conclusions

The activity of a chemical can be related directly to its structure. Activity prediction has incomparable advantages in terms of throughput, cost, and expandability for compounds in TCM [44]. Currently, attempts have been made to link computational activity prediction of TCM with modern QSAR methods [45]. In our study, multiple linear regression methods cannot be used to build the model since the number of variables is 10, and the number of independent variables of the 11 samples, especially for natural active ingredients, is less than 15. The PLS method contains further information for the maximum correlation between the dependent variable and extracted components and is suitable for small sample analysis. Thus, the PLS method was applied to build the QSAR model in our study of the diterpenoid alkaloid compounds based on their anti-inflammatory activities. The PLS-QSAR model displayed a good fit with the experimental data, with *R*^2^ = 0.981 and *Q*^2^ = 0.925. The prediction ability of the model was tested by four others as a forecasting dataset which showed good predictive power (*R*^2^*_pred_* = 0.8320). Applicability domain of the model covers all of the compounds. Moreover, anti-inflammatory activities of three unknown compounds (i.e., delsoline, fuziline, and songorine) were correctly predicted using the QSAR model. The prediction conclusion based on the model was consistent with the experiment results and the molecular docking results. In summary, our results indicate that QSAR based on the PLS method can improve quality control and risk assessment of TCM products. The activity of the diterpenoid alkaloids was closely related to the structure, and the model was capable of predicting the activity. These findings provide important information for further structure modification and discovery of new potential diterpenoid alkaloids with anti-inflammatory activities.

## Figures and Tables

**Table 1:** Predicted and experimental values of four diterpenoid alkaloids.

Compound ID	Name	Logarithm of *EC*_50_	Predicted Value	*R*^2^*_pred_*
**4**	Benzoylmesaconine	1.6989	0.9903	0.8320
**9**	Benzoyldeoxyaconine	0	−0.7386	
**12**	Benzoylaconine	0	0.2778	
**13**	Aconine	1.6989	2.6849	

**Table 2:** Inhibition rate (%) of 4 compounds at different concentrations on NO production by LPS-activated N9 cells (Mean ± SEM).

Compounds	1 μM	10 μM	30 μM	100 μM
Delsoline	118.06 ± 5.91	117.42 ± 1.29	112.26 ± 1.12	97.42 ± 3.10
Fuziling	117.42 ± 3.42	103.23 ± 3.24	127.74 ± 3.55	110.32 ± 3.52
Songorine	110.97 ± 3.96	93.55 ± 6.35	70.97 ± 3.41	58.71 ± 0.65
Minocycline ^a^	91.67 ± 4.27	88.73 ± 3.22	73.04 ± 2.45	40.20 ± 3.07

^a^ Minocycline was used as the positive control.

**Table 3:** The chemical structures of 15 diterpenoid alkaloids and Log(*EC*_50_) values.

NO.	Compounds	R1	R2	R3	R4	R5	R6	Log(*EC*_50_)
1	Deoxyaconitine	CH_3_CH_2_	CH_3_COO	H	C_6_H_5_COO	OH		−0.699
2	Hypaconitine	CH_3_	OH	H	C_6_H_5_COO	OH		−0.301
3	Aconitine	CH_3_CH_2_	CH_3_COO	OH	C_6_H_5_COO	OH		−0.301
4 *	Benzoylmesaconine	CH_3_	OH	OH	C_6_H_5_COO	OH		1.699
5	Mesaconitine	CH_3_	CH_3_COO	OH	C_6_H_5_COO	OH		−0.301
6	Benzoylhypaconine	CH_3_	OH	H	C_6_H_5_COO	OH		2
7	3-Acetylaconitine	CH_3_CH_2_	CH_3_COO	CH_3_COO	C_6_H_5_COO	OH		−1
8	Bulleyaconitine	CH_3_CH_2_	CH_3_COO	H	OOCC_6_H_4_OCH_3_	OH		−0.699
9 *	Benzoyldeoxyaconine	CH_3_CH_2_	OH	H	C_6_H_5_COO	OH		0
10	Yunaconitine	CH_3_CH_2_	CH_3_COO	OH	OOCC_6_H_4_OCH_3_	H		1.301
11	Ignavine							2
12 *	Benzoylaconine	CH_3_CH_2_	OH	OH	C_6_H_5_COO	OH		0
13 *	Aconine	CH_3_CH_2_	OH	OH	OH	OH		1.699
14	Lappaconine						NHCOCH_3_	0.778
15	*N*-Deacetyllappaconitine						NH_2_	1.176

* NO. 4, 9, 12, 13 were used as a forecasting dataset.

## Supplementary Materials

Supplementary materials are available online.
