# Supplementary material for: An Investigation on the Quantitative Structure-Activity Relationships of the Anti-Inflammatory Activity of Diterpenoid Alkaloids
Source: Molecules. 2017 Feb 27;22(3):363. doi: 10.3390/molecules22030363 (PMC6155234; doi:10.3390/molecules22030363)
Supplement: Supplementary file 1 [file molecules-22-00363-s001.pdf]

Supplementary Materials: The Investigation on Quantitative Structure-Activity Relationship of the Anti-Inflammatory Activity for Diterpenoid Alkaloids

Xiao Li, Ning Li, Zhenyu Sui, Kaishun Bi and Zuojing Li

Table S1. The weights for the descriptors and the responders for the three components in the loading plot of PLS-weight in the PLS model.

Variable	Component 1	Component 2	Component3
SAA	0.08536	−0.3313	−0.04633
SAG	0.06451	−0.2963	0.2549
VOL	0.04399	−0.29608	0.2690
HE	−0.2246	0.07921	0.1940
LogP	−0.05427	−0.4194	−0.8050
REF	−0.5720	−0.6755	−0.6162
POL	−0.5076	−0.1768	0.1534
MASS	−0.4082	0.03622	0.2199
BE	0.1623	−0.3474	0.05742
HF	0.3942	−0.1200	−0.1673
$logEC_{50}$	0.5034	0.2134	0.1530

Table S2. The predicted anti-inflammatory activities by PLS-QSAR model and the experiment anti-inflammatory activities and for 3 diterpenoid alkaloids.

Compound Name	Predicted Value (EC_{50})	Experiment Value
songorine	e3.8805	exhibiting inhibitory effect
fuziling	e4.0517	exhibiting no inhibitory effect
delsoline	e0.3014	exhibiting no inhibitory effect

Greater value of EC_{50} indicates lower activity of the compound. The values of EC_{50} $e^{3.8805}$ and $e^{4.0517}$ do not indicate activities for fuziline and delsoline.
